# Intraspecific genetic variation of the hairy-fruited eggplant (*Solanum lasiocarpum* Dunal.) based on plastid genome sequences

**DOI:** 10.1038/s41598-025-33167-4

**Published:** 2026-01-23

**Authors:** Rebecca Jia Yiin Ng, Sanit Kaewdaungdee, Yangyang Liu, Arunrat Chaveerach, Xue Jing Wong, Chuan Guo, Douglas Sie Nguong Law, Shiou Yih Lee

**Affiliations:** 1 Faculty of Health and Life Sciences, INTI International University, Nilai, 71800 Negeri Sembilan Malaysia; 2 Department of Biology, Faculty of Science, Khon Kaen University, Khon Kaen, 40002 Thailand; 3 Hainan Branch Institute of Medicinal Plant Development, Chinese Academy of Medical Sciences and Peking Union Medical College/ Hainan Key Laboratory of Resources Conservation and Development of Southern Medicine/ International Joint Research Center for Quality of Traditional Chinese Medicine, Haikou, 570311 Hainan China; 4 Faculty of Liberal Arts, Shinawatra University, Pathum Thani, 12160 Thailand; 5 Centre for Health, Well-being, and Environmental Sustainability, INTI International University, Nilai, 71800 Negeri Sembilan Malaysia

**Keywords:** Genome skimming, Genetic resources, Leptostemonum, Solanaceae, Terung asam, Evolutionary genetics, Phylogenetics

## Abstract

*Solanum lasiocarpum* Dunal. is a medicinal plant with medicinal properties for minor human diseases. To generate additional genomic resources for this species, we sequenced and characterised plastomes from seven *S. lasiocarpum* accessions collected in Malaysia, Indonesia, and Thailand. The seven plastomes range from 155,616 to 156,854 bp and each contains 128 genes. We identified 43–46 simple sequence repeats and 32–37 long repeats per plastome, with a strong bias towards A/T-rich motifs. Two indels and three highly variable regions (*pet*A, *pet*A–*psb*J, *rpl*32–*trn*L-UAG) were detected. The intraspecific pairwise distance between the previously published Hainan accession, SLHN (GenBank accession no.: PP234975), and the seven new accessions was 0.001. Phylogenetic analyses based on both complete plastomes and protein-coding sequences (CDS) recovered SLHN as the earliest-diverging lineage, followed by a split into two major clades. In the plastome-tree, the Mantin (SLMT), Serian (SLSR), and Phukhieo (SLTH) accessions formed one clade, whereas the Lachau (SLLC), Pontianak (SLPT), Sibu (SLSB), and Sibu wild (SLSW) accessions grouped together. In the CDS-tree, SLMT and SLTH clustered together, separate from the SLLC + SLPT + SLSR + SLSB + SLSW clade. Most internal branches received low statistical support in both trees. These results provide new insights into intraspecific plastome variation in *S. lasiocarpum* and establish a comparative framework to support marker development, molecular breeding, and future phylogenetic and evolutionary studies in *Solanum*.

## Introduction

*Solanum* is the largest genus in the family Solanaceae, comprising more than 1,200 species^1^. Its taxonomic classification is notoriously complex; despite extensive efforts using both morphological and molecular data, many relationships remain unresolved^2–4^. A recent phylogeny based on a combined dataset of two nuclear and seven plastid regions from 742 *Solanum* taxa recognises three major clades—Clade I, Clade II, and Thelopodium. Within Clade I, two subclades are recovered: the M clade, which includes three groups (DulMo, Regmandra, VANAns), and the Potato subclade, which includes six groups (Anarrhichomenum, Basarthrum, Euberosum, Petota, Pteroidea–Herpystichum, Tomato). Clade II comprises seven subclades: Brevantherum, Cyphomandra, Germinata, Leptostemonum, Nemorense, Wendlandii–Allophyllum, and *S. anomalestemon*. The Leptostemonum subclade is further divided into 12 sections: Acanthophora, Androceras, Crinitum, Crotonoides, Eastern Hemisphere spiny, Elaeagnifolium, Erythrotrichum, Gardneri, Lasiocarpa, Micracantha, Thomasiifolium, and Torva^5^.

**Fig. 1:**
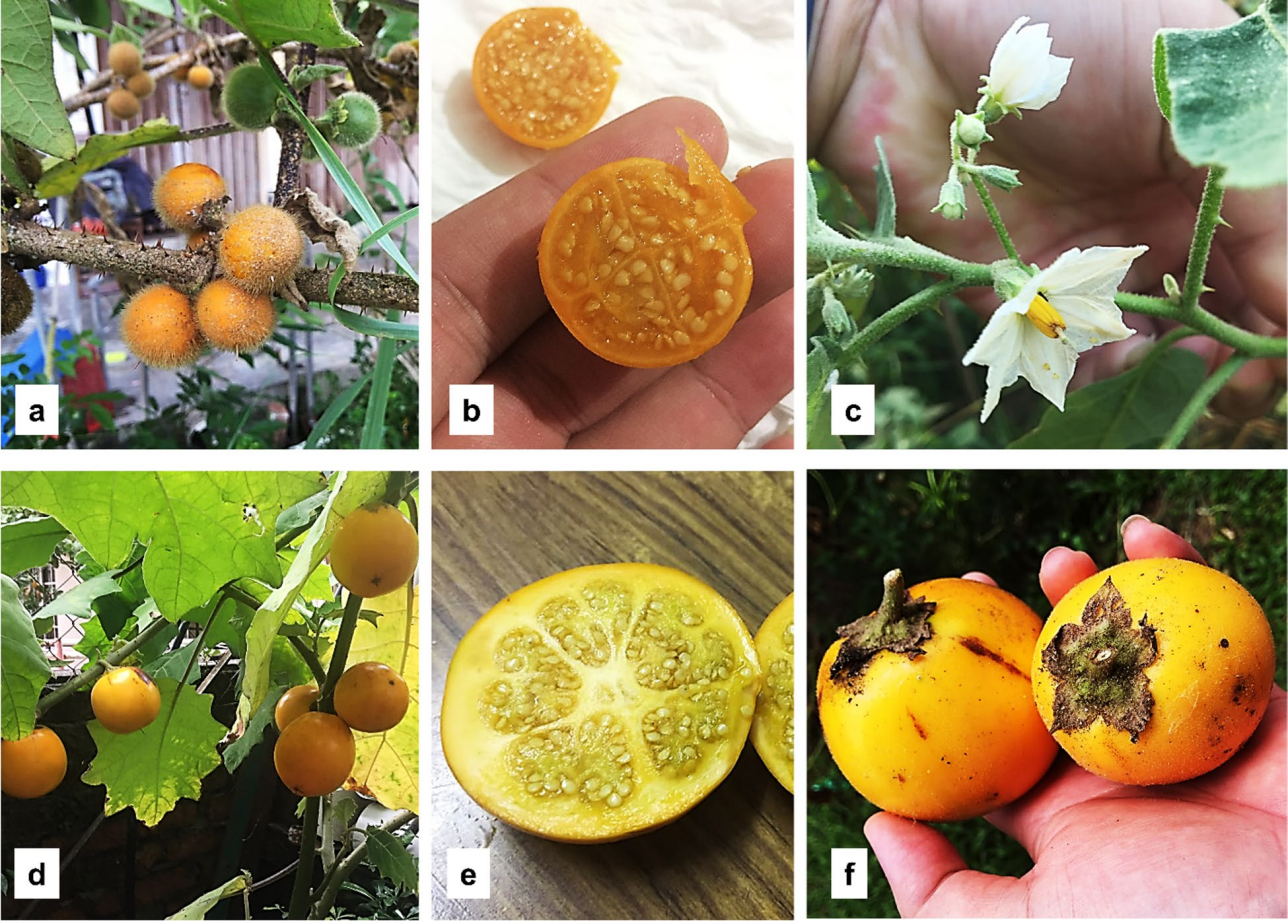
*Solanum lasiocarpum* from Mantin (SLMT; **a**–**c**) and Serian (SLSR; **d**–**f**). (**a**) 1–5 small hairy fruits attached on the thorny stem, (**b**) cross-sectional view of the fruit, (**c**) flower, (**d**) 1–3 big smooth fruits attached on the thorny stem, (**e**) cross-sectional view of the fruit, (**f**) size of the fruit on hand.

*Solanum lasiocarpum* of Solanaceae is placed in the Leptostemonum clade^6^. Cultivation is typically from seed, and breeding for varietal improvement has been rarely reported despite its widespread use in Malaysia, China, Thailand, and Indonesia^7^. It is grown for its distinctive flavour in local cuisines and is used in folk medicine to treat ailments such as coughs and skin irritations^8^. In the Borneo region, the fruit is recognised as a substitute for tomato and tamarind in cooking. Morphologically, *S. lasiocarpum* has erect, spreading, prickly, stellate-pubescent stems and globose fruits that ripen to a bright orange colour with juicy, sweet–sour flesh (Fig. 1)^9^. Fruits are 2.5–3.5 cm in diameter^8^ and occur in clusters of 1–10 per infructescence^9^. As a fruit-vegetable crop, it can yield approximately 16–20 t ha^−1^ under cultivation, particularly in larger-fruited forms^10^.

The taxonomy of *S. lasiocarpum* has long been problematic. Early classifications treated *S. ferox* as conspecific with *S. lasiocarpum*^11^, but the former is now treated as a rejected name^12^. A larger-fruited form was later described as *S. lasiocarpum* var. *domesticum*^13,14^ and subsequently synonymised under *S. lasiocarpum*. The original description of *S. lasiocarpum* did not specify fruit size^15^. As the fruit size alone is not a reliable diagnostic character, the observed differences among accessions are interpreted as phenotypic variation between wild and cultivated forms rather than evidence of distinct taxa^16^. Although recent revisions have clarified the taxonomic placement of *S. lasiocarpum*^9^, further work incorporating genetic data is needed to corroborate and refine these conclusions.

The characterisation of the plastid genome (plastome) via next-generation sequencing and genome assembly is one of the most cost-effective and informative ways to reveal the genomic information of an understudied plant species at the species level^17^. Generally, all plastomes have a quadripartite structure consisting of one large single copy, one small single copy, and two inverted repeats^18^. Due to its highly conserved nature when compared to the nuclear genome, the maternal inheritance of the plastome sequence makes it suitable for evolutionary studies, DNA barcoding, and genetic engineering^19^. Given the importance of *Solanum* as a food crop, ongoing efforts are focused on sequencing and characterising the plastomes of related *Solanum* species. However, for *S. lasiocarpum*, there is no published record thus far. Because the plastid is a center for biochemical processes even though it is highly conserved, the plastome sequence information could help the process of breeding for improved cultivar of *S. lasiocarpum*^17^.

Characterisation of the plastid genome (plastome) using next-generation sequencing and de novo assembly is a cost-effective and informative approach for recovering genomic information in understudied plant species^17^. Most plastomes exhibit a quadripartite architecture comprising a large single-copy (LSC) region, a small single-copy (SSC) region, and two inverted repeats (IRa and IRb)^18^. Owing to its high conservation relative to the nuclear genome and its predominantly maternal inheritance, the plastome is well suited to evolutionary studies, DNA barcoding, and genetic engineering^19^. Given the agronomic importance of *Solanum*, numerous efforts have focused on sequencing and characterising plastomes across the genus; however, for *S. lasiocarpum* the available data remain limited, with only a single publicly available plastome (SLHN; GenBank accession no. PP234975) prior to this study^20^. Although plastomes are highly conserved, plastids are central to key biochemical pathways; accordingly, plastome sequence information can inform marker development and support breeding for improved cultivars of *S. lasiocarpum*^17^.

Thus, in this study, we included seven *S. lasiocarpum* accessions sampled in Malaysia, Indonesia, and Thailand, to represent the main distribution range of the species in Southeast Asia. The selection covered both small, hairy-fruited and large, smooth-fruited forms that are commonly recognised by farmers and consumers. Six accessions were collected from cultivated stands, while one accession was obtained from a naturally occurring wild population. This sampling strategy was intended to capture the range of morphological and ecological variation observed in *S. lasiocarpum* and to examine whether plastome variation corresponds with cultivation status, fruit type, or geographical origin. These data enhance understanding of the species and provide a foundation for future breeding efforts.

## Results

### Plastome structure

**Fig. 2:**
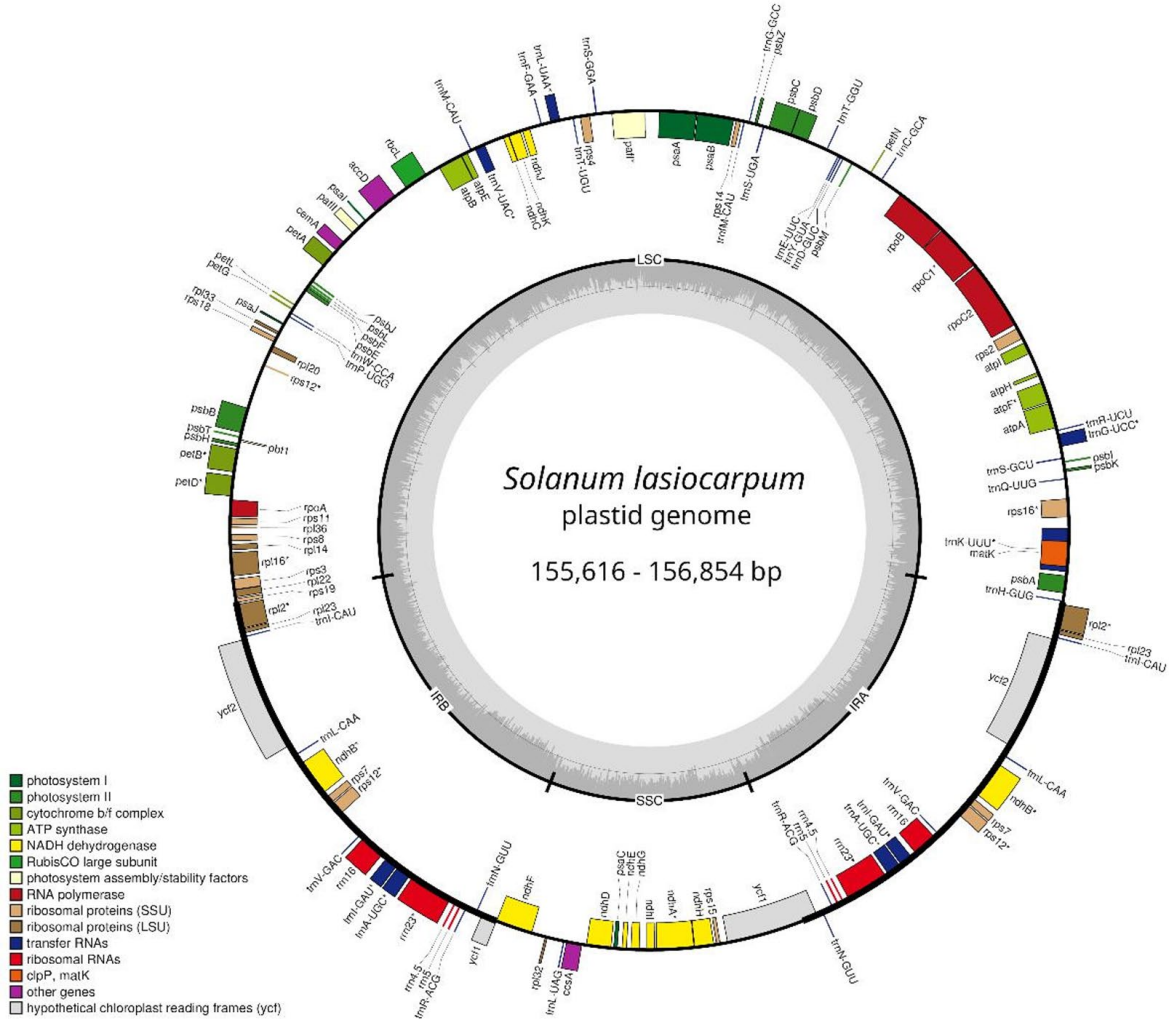
Plastid genome map of *Solanum lasiocarpum*. Genes annotated outside of the circular map are transcribed counterclockwise, while those inside the circular map are transcribed clockwise. Genes are colour-coded to indicate functional groups. Gray shading within the circle of the map indicated GC content. Genes with asterisk (*) contains introns.

The length of the complete plastome sequences of the seven *S. lasiocarpum* accessions assembled in this study (GenBank accession numbers: PV013415—PV013421) was in the range of 155,616 to 156,854 bp (Table 1; Fig. 2), with SLLC, SLPT, and SLSB having the shortest genome sequence (155,616 bp), while SLSR and SLTH had the longest sequence (156,854 bp). All plastomes had a typical quadripartite structure, including one large single-copy region (LSC; 86,291–87,529 bp) and one small single-copy region (SSC; 18,548–18,549 bp) that were separated by a pair of inverted repeat regions (IRs; each 25,388 bp). The overall GC content was 37.7%. A total of 128 genes were annotated in all plastomes, consisting of 83 CDS, 37 tRNA, and eight rRNA genes (Table 2). Among them, 18 genes were duplicated in the IR region, including seven protein-coding (i.e., *ndh*B, *rpl*2, *rpl*23, *rps*7, *rps*12, *ycf*1, and *ycf*2), seven tRNA (i.e., *trn*A-UGC, *trn*I-CAU, *trn*I-GAU, *trn*L-CAA, *trn*N-GUU, *trn*R-ACG, and *trn*V-GAC), and four rRNA (i.e., *rrn*4.5, *rrn*5, *rrn*16, and *rrn*23) genes. A total of 16 genes had one intron, including nine CDS genes (i.e., *atp*F, *pet*B, *pet*D, *rp1*16, *rpo*C1, *rps*16, *ndh*B, *ndh*A, and *rpl*2), six tRNA genes (i.e., *trn*A-UGC, *trn*G-UCC, *trn*I-GAU, *trn*K-UUU, *trn*L-UAA, and *trn*V-UAC), and the rRNA gene, *rrn*23. Two CDS genes were detected with two introns, i.e., *paf*I and *rps*12.

**Table 1:** Information of the origin and plastid genome characteristics of the seven *Solanum lasiocarpum* accessions sequenced in this study.

	SLLC	SLMT	SLPT	SLSB	SLSR	SLTH	SLSW
Sample of origin	Lachau of Sarawak, Malaysia	Mantin of Negeri Sembilan, Malaysia	Pontianak of Kalimantan, Indonesia	Sibu of Sarawak, Malaysia	Serian of Sarawak, Malaysia	Phukhieo of Chaiyaphum, Thailand	Sibu of Sarawak, Malaysia
Cultivated (C)/ Wild (W)	C	C	C	C	C	C	W
Size of fruit (B = big, S = small)	B	S	B	B	B	S	B
Collector; Collection number	S.Y.Lee; LSY16	S.Y.Lee; LSY15	S.Y.Lee; LSY18	X.J.Wong; WXJ23-001	S.Y.Lee; LSY09	S. Kaewdaungdee & A. Chaveerach; A.Chaveerach1109	S.Y.Lee; LSY17
Total genome size (bp)	155,616	155,617	155,616	155,616	156,854	156,854	155,617
Large single-copy (bp)	86,292	86,292	86,291	86,291	87,529	87,529	86,292
Small single-copy (bp)	18,548	18,549	18,549	18,549	18,549	18,549	18,549
Inverted repeat (bp)	25,388	25,388	25,388	25,388	25,288	25,388	25,388
GC content (%)	37.7	37.7	37.7	37.7	37.7	37.7	37.7
Number of protein-coding genes	83	83	83	83	83	83	83
Number of transfer RNA genes	37	37	37	37	37	37	37
Number of ribosomal RNA genes	8	8	8	8	8	8	8
GenBank accession no.	PV013415	PV013416	PV013417	PV013419	PV013418	PV013421	PV013420

**Table 2:** Genes annotated in the plastid genome of *Solanum lasiocarpum.* Genes that come in duplicates are indicated with an asterisk “*” behind the gene name.

Category	Gene Group	Name of gene
Photosynthesis related	Photosystem I	*psa*A, *psa*B, *psa*C, *psa*I, *psa*J
	Photosystem II	*psb*A, *psb*B, *psb*C *psb*D, *psb*E, *psb*F, *psb*H, *psb*I, *psb*J, *psb*K, *psb*L, *psb*M, *psb*T, *psb*Z
	ATP synthase	*atp*A, *atp*B, *atp*E, *atp*F, *atp*H, *atp*I
	NADH oxidoreductase	*ndh*A, *ndh*B*, *ndh*C, *ndh*D, *ndh*E, *ndh*F, *ndh*G, *ndh*H, *ndh*I, *ndh*J, *ndh*K
	Cytochrome b6/f complex	*pet*A, *pet*B, *pet*D, *pet*G, *pet*L, *pet*N
	RubisCO	*rbc*L
	Photosystem assembly factor	*paf*1, *paf*II
	Photosystem biogenesis factor	*pbf*1
Transcription and translation	Transfer RNAs	*trn*A-UGC*, *trn*C-GCA, *trn*D-GUC, *trn*E-UUC, *trn*F-GAA, *trn*fM-CAU, *trn*G-GCC, *trn*G-UCC, *trn*H-GUG, *trn*I-CAU*, *trn*I-GAU*, *trn*K-UUU, *trn*L-CAA*, *trn*L-UAA, *trn*L-UAG, *trn*M-CAU, *trn*N-GUU*, *trn*P-UGG, *trn*Q-UUG, *trn*R-ACG*, *trn*R-UCU, *trn*S-GCU, *trn*S-GGA, *trn*S-UGA, *trn*T-GGU, *trn*T-UGU, *trn*V-GAC*, *trn*V-UAC, *trn*W-CCA, *trn*Y-GUA
	Ribosomal RNAs	*rrn*4.5*, *rrn*5*, *rrn*16*, *rrn*23*
	Large subunit of ribosome	*rpl*2*, *rpl*14, *rpl*16, *rpl*20, *rpl*22, *rpl*23*, *rpl*32, *rpl*33, *rpl*36
	Small subunit of ribosome	*rps*2, *rps*3, *rps*4, *rps*7*, *rps*8, *rps*11, *rps*12, *rps*14, *rps*15, *rps*16, *rps*18, *rps*19
	DNA-dependent RNA polymerase	*rpo*A, *rpo*B, *rpo*C1, *rpo*C2
Biosynthesis	Maturase	*mat*K
	Envelope membrane protein	*cem*A
	C-type cytochrome synthesis	*ccs*A
	Acetyl-CoA-carboxylase	*acc*D
Unknown	Hypothetical chloroplast reading frames	*ycf*1*, *ycf*2*

### Simple sequence repeats (SSRs) and long repeat

**Fig. 3:**
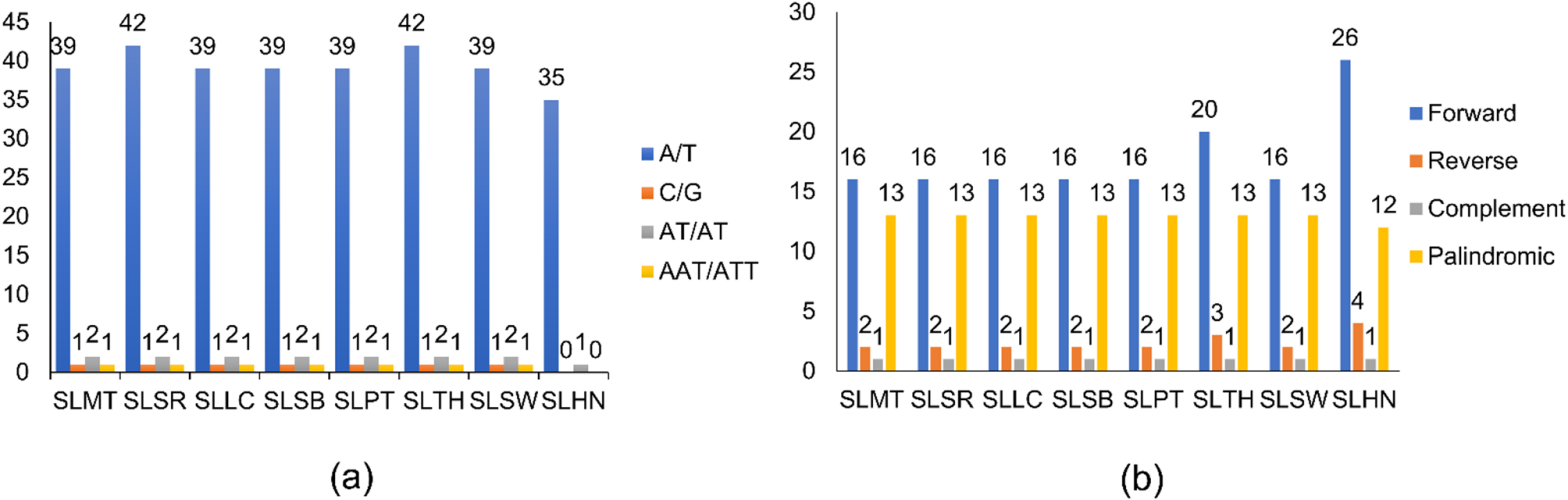
Repeat analyses of complete chloroplast genome sequence of different *Solanum lasiocarpum* accessions. (**a**) Different types of SSRs found (**b**) Different types of long repeats found.

The total number of SSRs found for the eight complete plastome sequences was between 36 and 46 (Fig. 3a). The most abundant was recorded for SLSR and SLTH, while the least abundant was recorded for SLHN. The other five complete plastome sequences were all recorded with 43 SSRs. All eight plastome sequences had a bias towards mononucleotide repeats, especially for the A/T motif, while all of them had one repeat on the C/G motif, except for SLHN. All plastome sequences were accounted for with two AT/AT repeats and one AAT/ATT repeat, except for SLHN, which had only one for the AT/AT motif, and no trinucleotide repeat was recorded. For the large repeats, a total of 32 to 43 large repeats were identified (Fig. 3b). For the forward repeat, SLHN had the highest count of 26, followed by SLSR and SLTH (*n* = 20). The others were recorded with 16 counts. For reverse repeat, SLHN had four counts, while SLSR and SLTH had three counts. The other plastome sequences were recorded with only two counts. All plastome sequences were recorded with one complement repeat and 13 palindromic repeats, except for SLHN, which only had 12 palindromic repeats.

### Inverted repeat border analysis

**Fig. 4:**
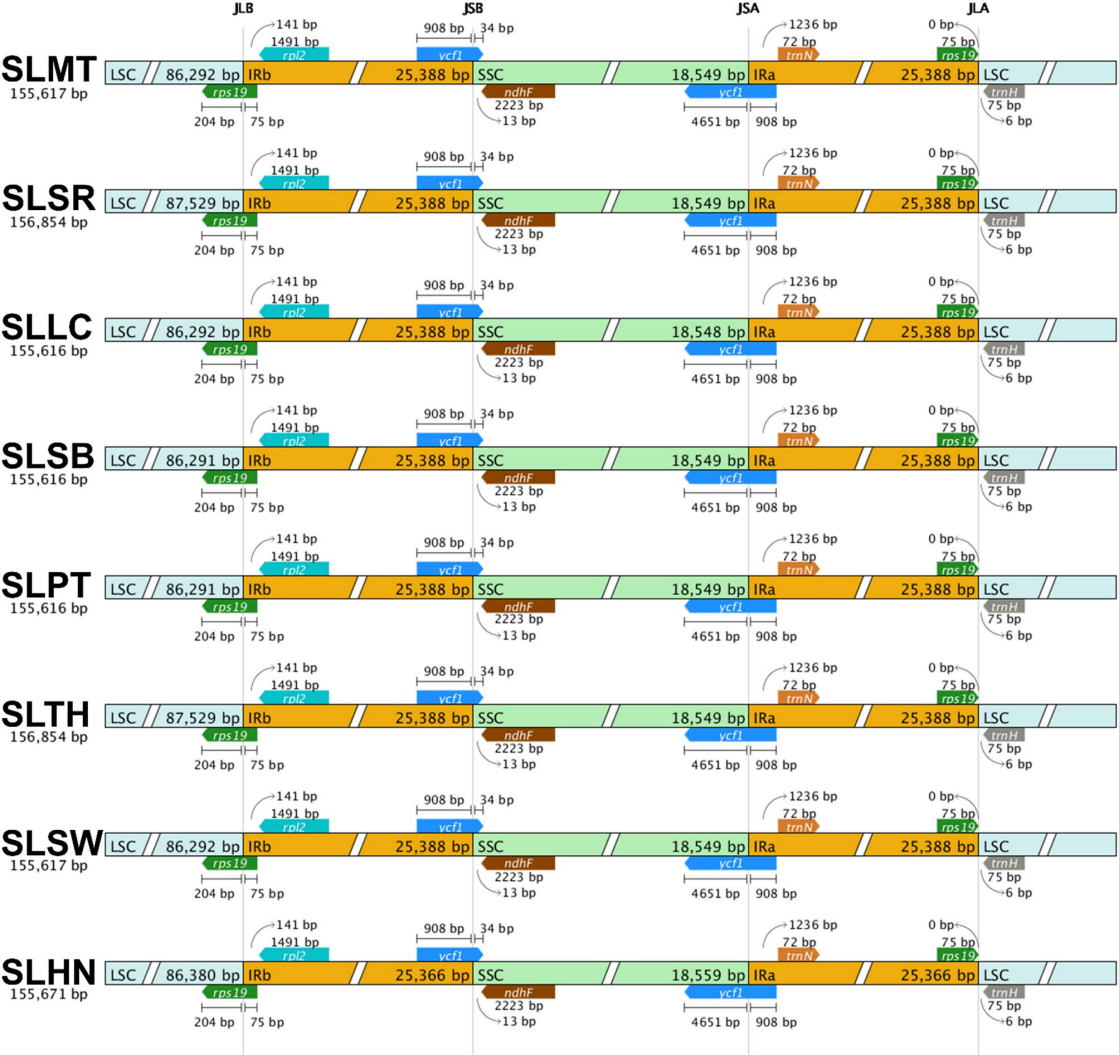
Comparison of eight *Solanum lasiocarpum* accessions’ complete plastid genome for the inverted repeat border regions.

All eight complete plastomes showed the same gene content adjacent to the IR borders (Fig. 4). At the junction between LSC and IRA (JLA), the *trn*H and *rpl*2 genes were placed in the LSC and IRA regions, respectively. At the junction between LSC and IRB (JLB), the *rps*19 gene was found crossing over from IRB into the LSC region. At the junctions between SSC and IRA (JSA) as well as SSC and IRB (JSB), the *ycf*1 genes were crossing over the junctions from the IR regions into the SSC region.

### Intraspecific nucleotide divergence

**Fig. 5:**
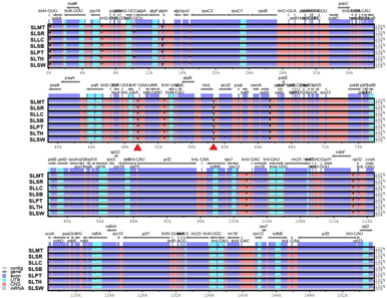
Comparison of nucleotide variation in the plastid genome sequence of seven different *Solanum lasiocarpum* accessions. The complete plastid genome sequence of Hainan accession (SLHN) was used as the reference sequence for comparison. The y-axis represents the percent identity within 50–100%. Grey arrows indicate the direction of gene transcription. Red arrows indicate the distinct gaps detected in the genome alignment.

When compared to the complete plastome of SLHN, only two distinct gaps were present in the plastome sequence of the other seven *S. lasiocarpum* samples (Fig. 5). The gaps were identified at the intergenic regions between *rbc*L and *acc*D genes, as well as *trn*L-UAA and *trn*F-GAA genes, which are caused by the 6-bp and 153-bp indel regions, respectively.

### Intraspecific pairwise distance and variation

The sequence alignment of the eight complete plastome sequences was 158,294 bp long. The pairwise distance between the eight *S. lasiocarpum* specimens was the same under pairwise deletion and complete deletion. The pairwise distance between SLHN and the other seven *S. lasiocarpum* was 0.001, while the pairwise distance within the seven *S. lasiocarpum* was 0.000 (Table 3). A total of 120 variable sites were identified in the sequence alignment, of which 118 were singletons and two were parsimony informative sites. The singletons only came in two variants, of which 77 were found in the LSC region, 29 in the SSC region, and 12 in the IR region. The parsimony informative sites also came with two variants, of which both were detected in the LSC and SSC regions.

**Table 3:** Intraspecific pairwise distance of the eight *Solanum lasiocarpum* specimens based on the complete plastid genome sequence under both pairwise deletion and complete deletion treatments.

	SLSW	SLMT	SLSB	SLSC	SLPT	SLTH	SLSR
SLMT	0.000						
SLSB	0.000	0.000					
SLLC	0.000	0.000	0.000				
SLPT	0.000	0.000	0.000	0.000			
SLTH	0.000	0.000	0.000	0.000	0.000		
SLSR	0.000	0.000	0.000	0.000	0.000	0.000	
SLHN	0.001	0.001	0.001	0.001	0.001	0.001	0.001

### Nucleotide diversity

**Fig. 6:**
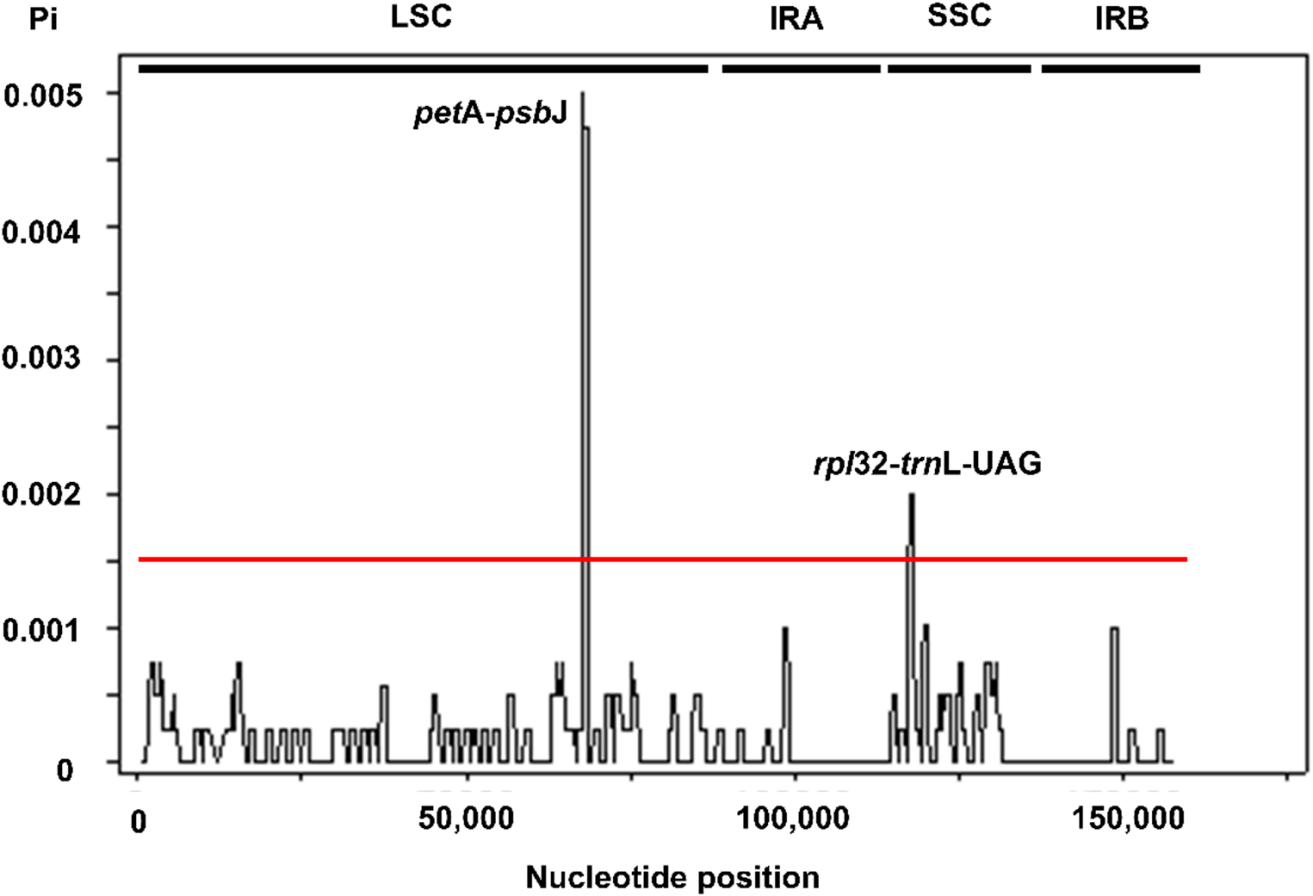
Nucleotide variability (Pi) analysis based on the complete plastid genome sequence alignment of seven *Solanum lasiocarpum* accessions, including the sequences constructed in this study.

By selecting the cutoff value for the nucleotide diversity, Pi = 0.0015, two highly divergent sites were found (Fig. 6). The first hotspot region was detected in the LSC region, encompassing the *pet*A gene and the intergenic spacer region *pet*A-*psb*J, while the second hotspot region was located at the intergenic spacer region *rpl*32-*trn*L-UAG in the SSC region.

### Phylogenetic inference

**Fig. 7:**
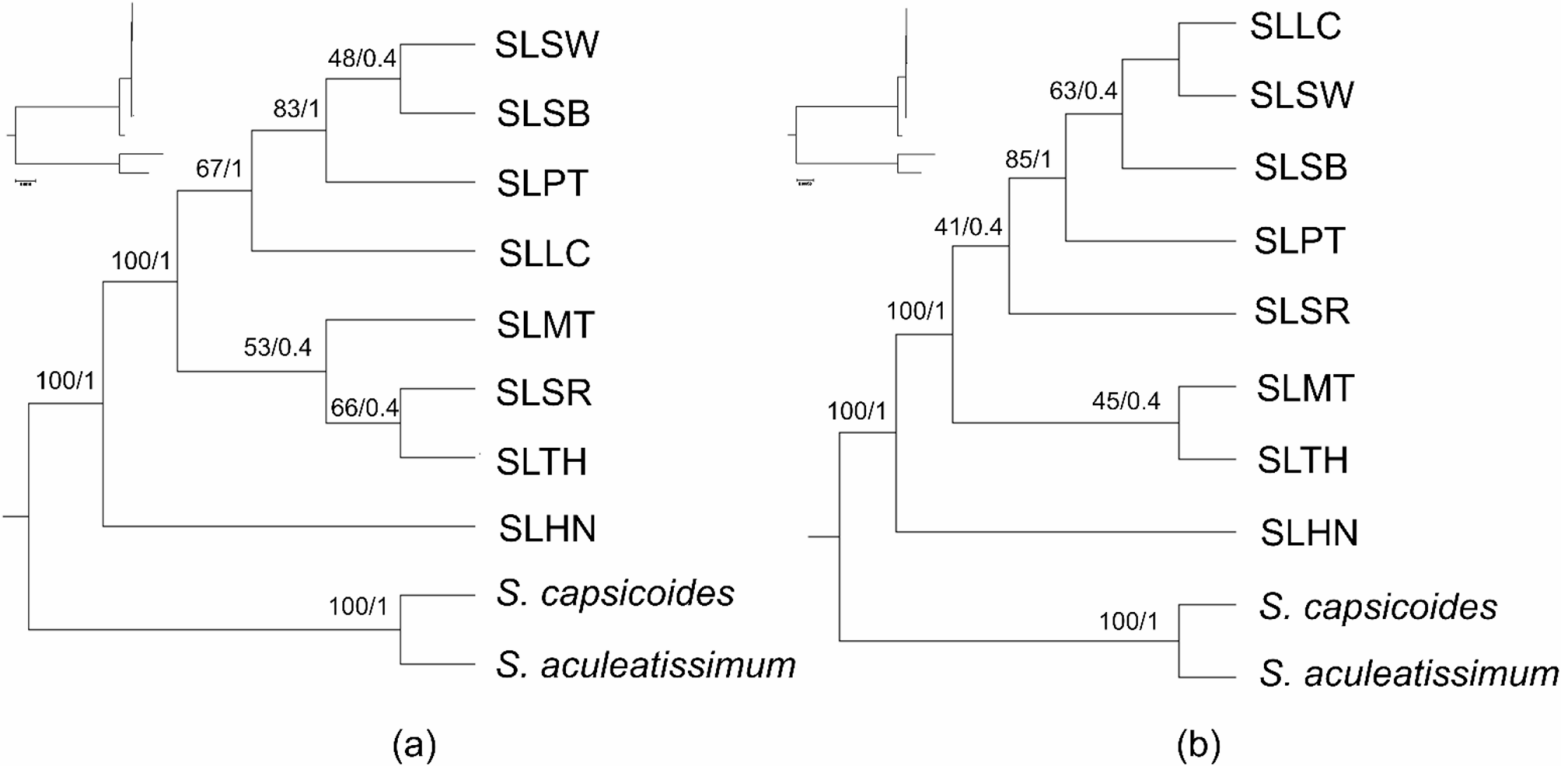
Phylogenetic analysis based on the eight selected *Solanum lasiocarpum* accessions using the (**a**) complete plastome sequence, with the IRA sequence excluded, and (**b**) concatenated dataset of 79 shared unique CDS. Two closely related species, *Solanum aculeatissimum* (GenBank accession no. OL679095) and *Solanum capsicoides* (GenBank accession no. MZ221890) were included as outgroup. A reliable branch support is indicated with an ultrafast bootstrap support value (left) of ≥ 95% and a posterior probability (right) of ≥ 0.95.

The phylogenetic tree reconstructed using the complete plastome and CDS datasets revealed a slightly different topology (Fig. 7). At the intraspecific level, SLHN was first to diverge from the other *S. lasiocarpum* samples in both the plastome- and CDS-tree. In the plastome-tree, the split between the SLMT + SLSR + SLTH clade and the SLLC + SLPT + SLSB + SLSW clade was well supported. However, within the SLMT + SLSR + SLTH clade, the divergence of SLMT from the other two accessions and the relationship between SLSR and SLTH, were not resolved. Within the SLLC + SLPT + SLSB + SLSW clade, SLLC was first to diverge, followed by SLPT, then SLSB and SLSW. However, the branch support for each accession was not well-supported under UFboot, but the divergence of SLLC and SLPT was supported under aBt. For the CDS-tree, the split between the SLMT + SLTH clade and the SLLC + SLPT + SLSB + SLSR + SLSW clade was well-supported. However, the relationship between SLMT and SLTH was unresolved. In the SLLC + SLPT + SLSB + SLSR + SLSW clade, SLSR was first to diverge, followed by SLPT, then SLSB, and lastly, SLLC and SLSW. In this clade, the divergence of all accessions was not well-supported under UFboot; only the divergence of SLPT was supported under aBt.

## Discussion

This is the first report analysing the intraspecific diversity of *S. lasiocarpum* using plastomes and CDS from at least four different countries. The genome size and gene content of the seven *S. lasiocarpum* accessions are conserved, and all shared the same number of genes. Genes that are adjacent to the IR junctions are the same within the species, similar to those of *S. aethiopicum*, *S. anguivi*, and *S. melongena*^21^. This also indicated that the evolution rate in the plastome region within *S. lasiocarpum* from different accessions is parallel across various locations. Diversity in genetic variation in SSRs and repeats is crucial for identifying molecular markers that can integrate desirable traits to create new cultivars^22^. In general, plastomes have a tendency for A/T repeats, as demonstrated in this study, where more than 90% of SSRs found were of A/T motifs^23^. The number of long repeats found was mostly forward, followed by palindrome, then reverse, and complementary repeats, which is consistent with the previous findings on *S. lycopersicum*^24^. When compared to the seven newly assembled plastome sequence, the plastome sequence of SLHN exhibited slight difference in terms of number of genes as well as number and types of repeat motifs. At the intraspecific level, we hypothesise that these differences may reflect population fragmentation and drift in SLHN.

The pairwise divergence between SLHN and the seven newly assembled *S. lasiocarpum* accessions is approximately 0.001 (per site), corresponding to roughly one SNP/indel per kilobase of plastome sequence. Although this intraspecific divergence exceeds that reported between two *Ipomoea obscura* (Convolvulaceae, Solanales) plastomes from China and Thailand (0.000421)^23^, in both species polymorphic sites are concentrated in the LSC and relatively depleted in the IR. Indels in the intergenic spacers *rbc*L–*acc*D and *trn*L-UAA–*trn*F-GAA account for part of the observed differences between SLHN and the other *S. lasiocarpum* accessions. SNP markers are crucial in breeding programmes for *Solanum* species, especially tomato and potato, where they inform assessments of genetic relationships and diversity, linkage map construction, gene discovery, and marker-assisted selection^25–27^. In a sliding-window analysis, *S. lycopersicum* exhibited more highly variable regions than *S. lasiocarpum*, with at least two genes and six intergenic spacers exceeding Pi = 0.002^26^, suggesting a faster plastome evolutionary rate in *S. lycopersicum*. In *S. lasiocarpum*, the few highly variable regions detected represent promising plastid markers for future phylogenetic, population-genetic, and phylogeographic studies.

Across our sampling, the *S. lasiocarpum* accessions segregate into two fruit morphotypes: small, round, pubescent fruits (SLMT, SLTH, SLHN) and large, elongate-round, smooth (glabrous) fruits (SLSR, SLLC, SLSB, SLSW, SLPT; see Fig. 1). Mapping these morphotypes onto the plastome phylogenetic trees yields a pattern congruent with the CDS-based tree: SLHN is the earliest-diverging lineage, whereas SLMT and SLTH form a clade separate from the larger-fruited accessions. Both trees indicate that all accessions share a common ancestor (i.e., *S. lasiocarpum* is monophyletic in our dataset). With the current sampling, the small-fruited lineage appears to predate the large-fruited lineage, consistent with the treatment of large-fruited *S. lasiocarpum* as a cultivar^28^. The weak branch support observed likely reflects limited outgroup representation and the paucity of available *Solanum* plastomes, which hampers identification of the immediate sister lineage, despite the Lasiocarpa section comprising at least 12 species^29^. *Solanum lasiocarpum* is thought to be closely related to *S. repandum* and *S. candidum*^30,31^, both of which produce fruits similar in size to the small-fruited *S. lasiocarpum* morphotype. We cannot exclude the possibility that large-fruited cultivars originated via hybridisation, with *S. lasiocarpum* as the maternal parent^14,32^. Testing this hypothesis will require nuclear-genome evidence; we therefore recommend reconstructing nuclear phylogenies using NGS-derived ribosomal DNA operon sequences or low-copy nuclear loci, approaches shown to be effective for resolving intraspecific variation in complex groups^33,34^.

**Fig. 8:**
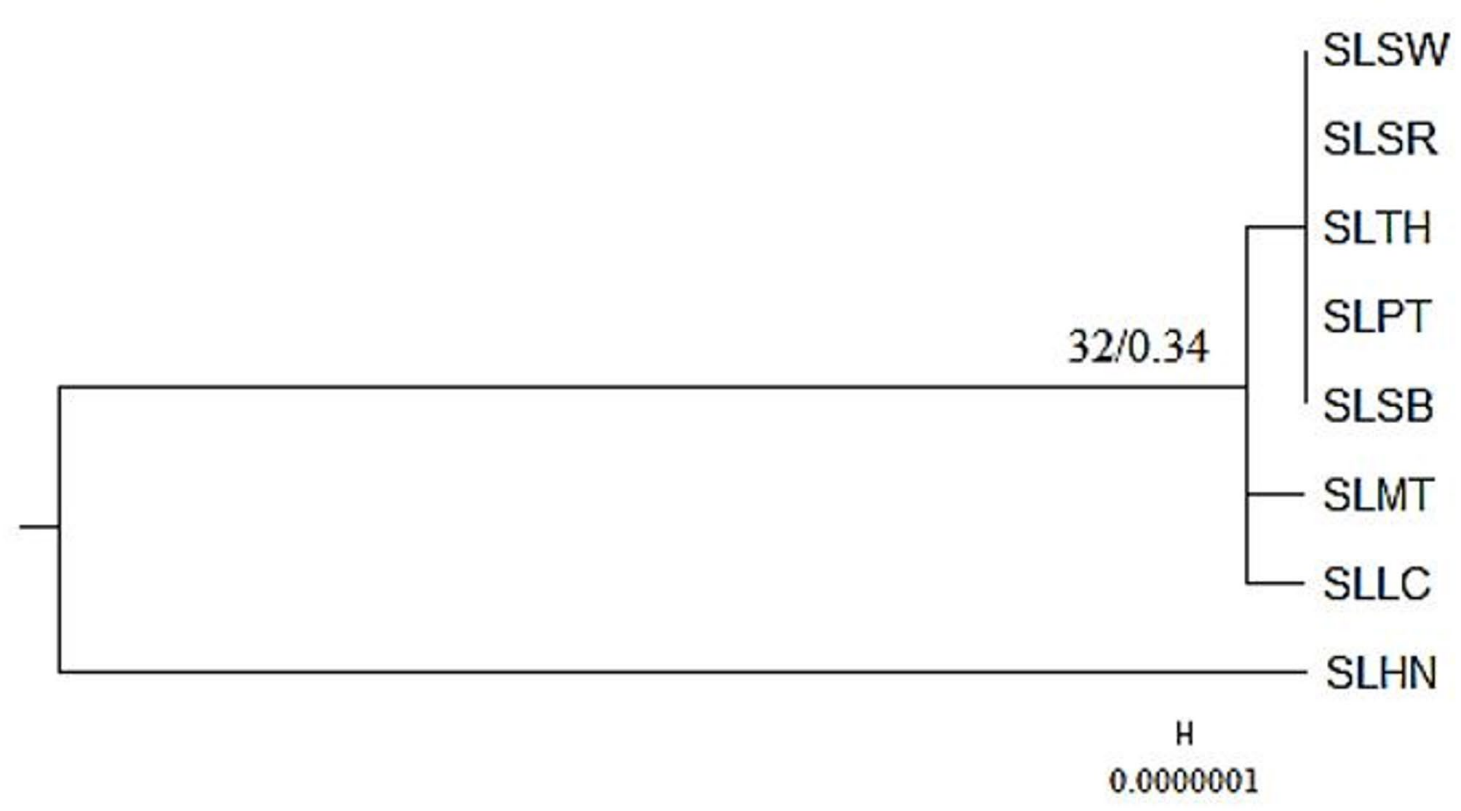
Phylogenetic analysis based on the eight selected *Solanum lasiocarpum* accessions using the two hotspot regions identified from the sliding window analysis (i.e., *pet*A-*psb*J and *rpl*32-*trn*L-UAG). The most optimum nucleotide substitution model was K3Pu + F. A reliable branch support is indicated with an ultrafast bootstrap support value (left) of ≥ 95% and a posterior probability (right) of ≥ 0.95. Branch support value that is calculated as 0 will not be shown.

An attempt on phylogenetic analysis was carried out using the two hotspot regions identified from the sliding-window analysis (i.e., *pet*A–*psb*J and *rpl*32–*trn*L-UAG). The concatenated alignment of these two regions was 3,634 bp in length and contained 28 variable sites. The resulting phylogenetic tree did not yield higher resolution or stronger branch support than the trees reconstructed from the complete plastome and CDS datasets. When rooted with SLHN, SLMT and SLLC formed one clade, while the remaining five accessions grouped into another; however, the separation between these clades was poorly supported, indicating that the two hotspot regions provided limited phylogenetic signal (Fig. 8). The overall topology also differed from that obtained using the complete plastome and CDS datasets. This discrepancy is likely due to the small number of informative sites within the selected regions, a limitation frequently observed in plastid-based phylogenetic trees of *Solanum* and other Solanaceae species^3,9,21,23,24^. Although these loci represent the most variable parts of the plastome, they constitute only a minor portion of the genome, providing insufficient data to resolve fine‐scale relationships. In contrast, analyses based on the complete plastome or concatenated CDS datasets incorporate a much larger number of characters, resulting in more stable tree topologies even when overall sequence variation is low^3,4^. These findings suggest that, while the hotspot regions may serve as useful molecular markers for population‐level studies, they are insufficient on their own for resolving intraspecific relationships in *S. lasiocarpum*.

In the context of domestication and plastome evolution in *S. lasiocarpum*, our comparative plastome analyses detected no divergence between cultivated and wild accessions. This outcome is consistent with (i) the generally high structural conservation of angiosperm plastomes and the limited intraspecific plastome variation reported at comparable scales^33,34^, and (ii) the taxonomic and historical context of *S. lasiocarpum*, including its placement within the spiny solanums and the domestication narrative surrounding larger-fruited forms formerly referred to as *S. lasiocarpum* var. *domesticum*^9,13,14^. The shared IR-junction architecture and broadly similar SSR/long-repeat profiles across cultivated and wild accessions further support shared maternal lineages. These patterns may reflect a recent domestication history, ongoing gene flow from wild populations into cultivated stocks, and/or selection acting primarily on the nuclear genome rather than the plastome. Discriminating among these non-mutually exclusive hypotheses will require broader geographic sampling and comparative analyses of nuclear markers alongside plastome data.

A recent large-scale plastome study on *Solanum* section Petota have shown that expanding intraspecific and interspecific sampling greatly improves the resolution and reliability of plastid-based phylogenetic trees^35^. Despite the current dataset represents a much smaller number of accessions, the seven newly sequenced *S. lasiocarpum* plastomes fill a major gap in genomic data for section Lasiocarpa within the Leptostemonum clade^21,23,24^. These data are useful for future comparative analyses and will help refine the evolutionary relationships among members of section Lasiocarpa after additional plastomes of closely-related species become available. Thus, our study not only lays the foundation for the phylogenetic expansion achieved in section Petota, but also supports ongoing efforts to resolve the evolutionary history of the spiny solanums.

### Methods

Fresh leaves of seven *S. lasiocarpum* accessions were collected. These accessions originate from cultivated stands in Mantin of Negeri Sembilan (SLMT), Serian of Sarawak (SLSR), Lachau of Sarawak (SLLC), Sibu of Sarawak (SLSB), Pontianak of Kalimantan, Indonesia (SLPT), and Phukhieo of Chaiyaphum, Thailand (SLTH), as well as a wild accession in Sibu of Sarawak (SLSW) (Table 1). All the accessions were planted in the Herb Garden of INTI International University for record purposes.

Total genomic DNA was extracted using the DNeasy Plant Mini Kit (Qiagen, USA) following the manufacturer’s protocol. The genomic DNA extracts were checked for their purity and concentration using a Qubit 4 fluorometer (Thermo Fisher Scientific, USA) before being sent for next-generation sequencing at Guangzhou Jierui Biotechnology Company, Ltd. (Guangzhou, China). A 350-bp paired-end genomic library was prepared using the TrueSeq DNA Sample Prep Kit (Illumina, USA), and 150-bp paired-end reads were sequenced using the NovaSeq 6000 platform (Illumina, USA). Total genomic DNA was extracted using the DNeasy Plant Mini Kit (Qiagen, USA) following the manufacturer’s protocol. The genomic DNA extracts were checked for their purity and concentration using a Qubit 4 fluorometer (Thermo Fisher Scientific, USA) before being sent for next-generation sequencing at Guangzhou Jierui Biotechnology Company, Ltd. (Guangzhou, China). A 350-bp paired-end genomic library was prepared using the TrueSeq DNA Sample Prep Kit (Illumina, USA), and 150-bp paired-end reads were sequenced using the NovaSeq 6000 platform (Illumina, USA). The complete plastome sequence was assembled by feeding the NGS raw data into the NOVOwrap v1.20^36^ software. The *rbc*L gene sequence of *Solanum candidum* (GenBank accession no. MH588527) was used as the seed sequence. Gene annotation was carried out using GeSeq v2.03^37^ and was manually checked for errors. The IR borders were verified using Geneious Prime v.2020.0.2^38^, and the physical map of the complete plastome was visualised using OGDraw v1.3.1^39^.

To provide a better comparison at the intraspecific level, together with the seven assembled plastome sequences, the complete plastome sequence of *S. lasiocarpum* from Hainan, China (SLHN; GenBank accession no. PP234975)^20^, was included in the subsequent analyses. Simple sequence repeats (SSRs) in the complete plastomes were calculated using the MISA-web^40^ online software based on a set of parameters for the minimum number of repeats. For mono-, di-, tri-, tetra-, penta-, and hexa-nucleotides, the parameters were set at 10, 6, 5, 5, 5, and 5 minimum repeats, respectively. Large repeats with a hamming distance of 3 and a minimum repeat length of 30 bp were identified through the REPuter programme^41^ for four different types of repeats, i.e., palindromic, forward, reverse, and complement repeats. The expansion and reduction of the IR borders of the complete plastomes were compared using CPJSdraw v1.0.0^42^. The complete plastome sequences were aligned using Shuffle-LAGAN mode. mVISTA^43^ was used to detect the intraspecific variation in the sequence alignment of the complete plastome sequences. The annotated plastome sequence of SLHN was selected as the reference genome. To calculate the intraspecific pairwise distance among the eight *S. lasiocarpum* accessions, the complete plastome sequences were aligned using MAFFT v.7^44^, and pairwise distances were calculated using MEGA7^45^ based on the Kimura two-parameter (K2P) DNA substitution model. Variance estimation was carried out using the bootstrap method, in which 1,000 bootstrap replicates were performed. The sequence alignment was analysed first including the gap and missing data (reflecting pairwise deletion, then analysed separately excluding the gaps and missing data (reflecting complete deletion). The variable and parsimonious sites, as well as the highly variable regions present in the sequence alignment, were detected using DnaSP v5.0^46^. A sliding window analysis was used to detect the hotspot spots in the complete plastome sequences, to which a window length of 1,000 bp and a step size of 200 bp were applied.

Phylogenetic analysis was conducted using the complete plastome sequences, with the sequence of the IRA region excluded, and the concatenated dataset of the protein-coding (CDS) region, of 10 *Solanum* taxa derived from eight *S. lasiocarpum* accessions. Given the limited genomic resources for the Lasiocarpa section of the Leptostemonum clade, and following Levin et al.^47^, we selected two closely related species from the sister Acanthophora clade, *S. aculeatissimum* (GenBank accession no.: OL679095) and *S. capsicoides* (GenBank accession no.: MZ221890), as outgroup taxa. Prior to phylogenetic tree reconstruction, for the complete plastome dataset, the sequences were aligned using MAFFT v.7^44^. For the CDS dataset, the 79 unique CDS regions in the complete plastome sequence were extracted using PhyloSuite v.1.2.3^48^, separately MAFFT-aligned, and concatenated using the plug-ins available in the PhyloSuite program. An edge-unlinked partition mode was selected for the CDS dataset. The optimum nucleotide substitution model for both datasets was assessed using the ModelFinder function embedded in the programme under the Bayesian inference criterion, of which the Kimura three substitution types model with unequal (K3Pu) and empirical base frequencies (+ F) with invariable sites included (+ I) (= K3Pu + F + I) model and the transversion model (TVM) with empirical base frequencies (+ F) (= TVM + F) model were identified as most suitable for the plastome and CDS datasets, respectively. The phylogenetic tree based on the maximum likelihood (ML) method was reconstructed using IQ-Tree v.1.6.8^49^ embedded in PhyloSuite^48^, in which the branch support was estimated using 1,000 replicates according to the ultrafast bootstrapping algorithm (UFboot) and approximate Bayesian test (aBt). The final tree result was visualised using FigTree v1.4.4^50^.

## Data Availability

The datasets generated and/or analysed during the current study are available in the NCBI GenBank repository, https://www.ncbi.nlm.nih.gov/, under the accession numbers PV013415 – PV013421.
